# Emerging Therapies in Cutaneous Lupus Erythematosus

**DOI:** 10.3389/fmed.2022.968323

**Published:** 2022-07-11

**Authors:** Grant Sprow, Joshua Dan, Joseph F. Merola, Victoria P. Werth

**Affiliations:** ^1^Dermatology, Perelman School of Medicine, University of Pennsylvania, Philadelphia, PA, United States; ^2^Dermatology, Corporal Michael J. Crescenz VA Medical Center, Philadelphia, PA, United States; ^3^Department of Dermatology, Department of Medicine, Division of Rheumatology, Brigham and Women's Hospital, Harvard Medical School, Boston, MA, United States

**Keywords:** cutaneous lupus erythematosus, autoimmune, skin, connective tissue disease, drug development

## Abstract

Cutaneous lupus erythematosus (CLE) is an autoimmune disease that can occur with or without underlying systemic lupus erythematosus (SLE) and often has a profoundly negative impact on patient quality of life. There is substantial need for new and more effective therapies to treat CLE. CLE has a multifactorial pathogenesis that involves several key immune cells and pathways, including abnormalities in innate (e.g., type 1 interferon pathways) and adaptive immune responses (e.g., B and T cell autoreactivity), presenting multiple opportunities for more targeted therapies that do not require immunosuppression. Here we review several emerging therapies and their efficacy in CLE. Anifrolumab and belimumab have both been approved for the treatment of SLE in recent years, and clinical trial evidence suggests some forms of CLE may improve with these agents. Therapies currently in development that are being evaluated with CLE-specific outcome measures include BIIB059 and VIB7734, which target plasmacytoid dendritic cells (pDCs), and iberdomide, a cereblon modulator. These novel therapies all have previously demonstrated clinical benefit in some forms of CLE. Other therapies which target molecules believed to play a role in CLE pathogenesis, such as Janus kinases (JAKs), spleen tyrosine kinase (SYK), interferon γ (IFNγ), IL-12, and IL-23, have been evaluated in lupus clinical trials with skin-specific outcomes but failed to meet their primary endpoints.

## Introduction

Systemic lupus erythematosus (SLE) is a complex autoimmune disease that can have various manifestations in the skin and internal organs. Most SLE patients develop cutaneous involvement at some point in the disease course which often has a significant impact on patient quality of life (1, 2). Cutaneous lupus erythematosus (CLE) is a set of heterogeneous inflammatory skin conditions with varying morphologies and scarring potential, that for the most part share common histopathologic features. CLE can occur with or without concomitant SLE.

There is substantial need for new therapies to treat CLE. Until very recently, there had been no new approved therapies for SLE since the 1950s. Few clinical trials have been designed to specifically evaluate therapies in CLE despite validated CLE-specific outcome measures such as the Cutaneous Lupus Erythematosus Disease Area and Severity Index (CLASI) (3). Quinacrine, a potential first-line therapy for CLE, is in short supply due to an import alert placed on the only manufacturer that once supplied the United States (4). Additionally, second line therapies often involve substantial immunosuppression, monitoring, and other safety concerns. Here, we review therapies recently approved for SLE, their efficacy/potential efficacy in CLE, as well as emerging therapies in development, and selected therapies that have been studied in CLE but ultimately failed in clinical trials.

## Therapies Approved for SLE

### Anifrolumab

Anifrolumab is a human, IgG1K monoclonal antibody that binds to type 1 interferon receptor, blocking type 1 interferon signaling. The scientific rationale for its mechanism of action is based on evidence indicating that the type 1 interferon pathway is involved in SLE pathogenesis (5). The FDA approved anifrolumab for SLE (though not lupus nephritis) in July 2021, the first new drug approval for SLE in over 10 years. After an initial phase 3 trial (TULIP-1) failed to meet its primary endpoint, a second phase 3 trial of anifrolumab (TULIP-2) showed that a higher proportion of patients in the treatment group had response at week 52 than those treated with placebo using a different primary endpoint than was used in TULIP-1 (6, 7). In TULIP-2, 362 patients were randomized to 48 weeks of treatment with either placebo or anifrolumab (6). Among patients with at least moderately severe skin disease, 49% of patients in the treatment group achieved the secondary endpoint of reduction in CLASI of 50% or greater compared to 25% in the placebo group, which was statistically significant (6). This data supports findings from TULIP-1 in which secondary endpoints pointed toward a clinical benefit in skin.

### Belimumab

Belimumab is a human monoclonal antibody that binds to soluble B-lymphocyte stimulator (BLyS). BlyS levels are commonly elevated in patients with SLE and correlate with increased disease activity (8–10). Binding of BLyS by belimumab leads to decreased survival of B cells and a reduction in the differentiation of B cells into antibody-producing plasma cells (11). Belimumab was the first biologic approved by the FDA for SLE in 2011 and was more recently approved for pediatric SLE in 2019 and lupus nephritis in 2020. Belimumab was studied in a phase 3 placebo-controlled trial of 819 randomized SLE patients and met its primary endpoint indicating a clinical benefit in reducing SLE disease activity (12). However, skin-specific outcomes were not included as endpoints in the study and so its efficacy in treating CLE was not initially evaluated (12). Using the rash component of the Systemic Lupus Erythematosus Disease Activity Index (SLEDAI), a *post-hoc* study of pooled phase 3 trial data showed approximately a 10% difference in two treatment doses relative to placebo after 52 weeks (13). This analysis also showed that significant improvements of two to three letter scores in the British Isles Lupus Assessment Group (BILAG), a lupus scoring system, were noted in the 10 mg/kg group in the mucocutaneous domain but not in the 1 mg/kg group (13). The change in overall adjusted mean SLEDAI scores from weeks 24 to 52 were significantly better with belimumab vs. placebo, indicating a potentially delayed effect across organ systems (13). Additionally, a number of case series have shown a significant reduction in CLASI-activity (CLASI-A) scores with the use of belimumab indicating a possible benefit (14–16).

## Therapies in Development

### Therapies Targeting Plasmacytoid Dendritic Cells

Plasmacytoid dendritic cells (pDCs) are considered one of the most crucial immune cells driving CLE pathogenesis. These cells are found in high numbers in CLE tissue after sun exposure and secrete pro-inflammatory cytokines and chemokines which drive disease progression (17). Two therapies targeting pDCs are currently in development for CLE.

BIIB059 is a humanized IgG1 monoclonal antibody targeting blood dendritic cell antigen 2 (BDCA2), a cell surface protein found exclusively on pDCs. This binding leads to inhibition of the production of pro-inflammatory mediators, including type 1 interferons, as well as decreased levels overall of inflammatory cells in patient tissue (18, 19). In a recent phase 2 trial of 132 patients with CLE, BIIB059 met its primary endpoint with a statistically significant difference in percent change from baseline in CLASI-A score compared to placebo (18). BIIB059 is currently being further evaluated in the phase 3 SLE trial TOPAZ-1 (ClinicalTrials.gov Identifier NCT04895241).

VIB7734 is another monoclonal antibody targeting pDCs currently being studied in patients with CLE. This antibody targets immunoglobulin-like transcript 7, a pDC-specific marker, and mediates depletion of pDCs through antibody-dependent cellular cytotoxicity (20). VIB7734 has been studied in two phase 1 trials and led to a reduction of circulating and tissue-resident pDCs in patients with CLE (20). Most CLE patients experienced clinical benefit as shown by reductions in CLASI-A scores (20). VIB7734 is currently being further studied in a phase 2 trial (ClinicalTrials.gov Identifier NCT04925934).

### Therapies Targeting Cereblon

IKZF1 and IKZF3 are susceptibility loci for SLE and encode the transcription factors Ikaros and Ailos (21). Cereblon is a molecule that forms part of a ubiquitin ligase complex which mediates the polyubiquitination and proteasome-dependent degradation of Ikaros and Ailos (22–24). Thalidomide and lenalidomide, drugs that have been used for off-label treatment of CLE, bind to cereblon resulting in increased destruction of Ikaros and Ailos (22–24), leading to decreased B cells, pDCs, and increased T regulatory cells (25, 26). Iberdomide (CC-220) is an oral compound in development for SLE that has been shown to have higher binding affinity for cereblon than lenalidomide resulting in increased Ikaros and Ailos degradation (27). This allows for the use of lower doses, lowering the potential for off-target effects.

Iberdomide did not show a significant difference from placebo in achieving a 50% or more reduction in CLASI-A score in a recent phase 2 trial when including all patients with a baseline CLASI-A score of at least 10 (28). However, the majority of patients in the trial had acute CLE (ACLE) (29). Further analysis by CLE subtype showed that patients with subacute CLE (SCLE) and chronic CLE (CCLE) were more likely to have a reduction in CLASI-A score of 50% or more when treated with iberdomide 0.45 mg than placebo (29). Additional research is needed to determine the true efficacy of iberdomide in treating CLE and its various subtypes.

## Therapies that Failed in Clinical Trials

### JAK and SYK Inhibitors

Janus kinases (JAKs) are involved in the signaling of several inflammatory cytokines that drive the pathogenesis of SLE (30–32). JAK/STAT signaling pathways are also upregulated within lesional CLE skin (33). Baricitinib is an oral selective and reversible inhibitor of JAK1 and JAK2 which has been studied as a potential treatment for SLE. In a phase 2 placebo-controlled trial of 314 SLE patients, baricitinib was found to be superior to placebo with standard of care in treating arthritis and nephritis (34). Improvement in skin disease was assessed as an outcome, but did not show significant difference from the placebo group (34). While many of the SLE patients had cutaneous involvement, the baseline activity scores were low which can make it difficult to demonstrate improvement (35). Baricitinib was also being evaluated in two phase 3 SLE trials, but top-line results led to the decision to discontinue the phase 3 development program in lupus. However, a phase 2 study of topical ruxolitinib, a JAK inhibitor, in discoid lupus erythematosus (DLE) is in development (ClinicalTrials.gov Identifier NCT04908280).

Spleen tyrosine kinase (SYK) activates pathways that increase inflammatory cytokine levels (36). Upregulated SYK activity has been seen in CLE skin and blocking SYK leads to decreased inflammatory cytokine levels in keratinocytes *in vitro* (37). Given the apparent roles of JAK1 and SYK in CLE pathogenesis, a randomized, placebo-controlled phase 2 trial comparing filgotinib, a JAK1 inhibitor, and lanraplenib, a SYK inhibitor, each to placebo was conducted but failed to meet the primary endpoint (38). A topical SYK inhibitor was also studied in a phase 1B trial but failed to show a difference from placebo in skin-specific disease measurement outcomes, perhaps due to low baseline disease activity (39). Another study of a topical JAK/SYK inhibitor had similarly negative results (40).

### AMG 811

AMG 811 is a human IgG1 anti-interferon γ (IFNγ) antibody with selectivity for human IFNγ (41). IFNγ is involved in the function of macrophages, B cells, and T cells, and its mRNA is found in higher levels in DLE skin than in normal skin (42). AMG 811 had several trials in patients with SLE which demonstrated acceptable safety and tolerability (43–45). However, a randomized phase 1 trial of 16 patients with DLE failed to show clinical efficacy in improving skin lesions compared to placebo (46).

### Ustekinumab

Ustekinumab is a human monoclonal antibody that blocks the p40 subunit of IL-12 and IL-23 and has FDA approval for the treatment of plaque psoriasis, psoriatic arthritis, Crohn's disease, and ulcerative colitis. IL-12 and IL-23 are important mediators of immunity and have been found in increased concentrations in patients with SLE (47, 48). A randomized phase 2 trial of 102 patients with SLE comparing ustekinumab to placebo plus standard therapy met its primary endpoint demonstrating a reduction in SLE disease activity (49). This trial also showed that patients in the ustekinumab group were more likely to achieve a 50% or greater reduction in CLASI-A score than patients in the placebo group, indicating a potential benefit in CLE (49). In a two-year open-label extension of this study, clinical benefits as measured by skin-specific and overall SLE activity outcomes were also seen (50). However, a phase 3 randomized, placebo-controlled trial of ustekinumab in SLE was halted due to lack of efficacy established during the interim analysis.

## Conclusion

Emerging therapies with clinical trial evidence demonstrating efficacy in some forms of CLE include anifrolumab, BIIB059, VIB7734, and iberdomide. Other pathways involved in lupus pathogenesis, such as tyrosine kinase 2 (TYK2) and serine/threonine kinase IL-1R–associated kinase (IRAK4), represent potential future therapeutic targets of interest. When designing clinical trials to evaluate therapies for potential use in CLE, careful consideration should be paid toward study design to allow for optimal chances of demonstrating clinical benefit; this includes enrolling a sufficient number of patients with moderate to severe baseline disease activity, as highlighted by the skin-specific data from the baricitinib phase 2 and topical SYK inhibitor phase 1B trials. Trials of SLE therapies should also include skin-specific outcome measures as CLE and SLE often demonstrate disparate responses to therapies.

## Author Contributions

GS, JD, and JM drafted the manuscript. VW supervised the work and revised the manuscript. All authors contributed to the article and approved the submitted version.

## Funding

United States Department of Veterans Affairs (Veterans Health Administration, Office of Research and Development and Biomedical Laboratory Research and Development), Lupus Research Alliance, DOD LR1901087, and National Institute of Health [R01AR071653].

## Conflict of Interest

VW has received grants from Celgene, Amgen, Janssen, Biogen, Gilead, Viela, Horizon therapeutics and has consulted for Astra-Zeneca, Pfizer, Biogen, Celgene, Resolve, Janssen, Gilead, Lilly, BMS, Nektar, Abbvie, Viela, GSK, EMD Serona, Sanofi, Anaptysbio, Amgen, Merck, Kyowa Kirin. JM has worked as a consultant and/or investigator for Amgen, Bristol-Myers Squibb, Abbvie, Dermavant, Eli Lilly, Novartis, Janssen, UCB, Sanofi,Regeneron, Sun Pharma, Biogen, Pfizer and Leo Pharma. The remaining authors declare that the research was conducted in the absence of any commercial or financial relationships that could be construed as a potential conflict of interest.

## Publisher's Note

All claims expressed in this article are solely those of the authors and do not necessarily represent those of their affiliated organizations, or those of the publisher, the editors and the reviewers. Any product that may be evaluated in this article, or claim that may be made by its manufacturer, is not guaranteed or endorsed by the publisher.
